# Differential contribution of A-type potassium currents in shaping neuronal responses to synaptic input

**DOI:** 10.1186/1471-2202-12-S1-P147

**Published:** 2011-07-18

**Authors:** Adrian Smith, Maytee Cruz-Aponte, Erin C McKiernan, Sharon Crook, Marco Herrera-Valdez

**Affiliations:** 1 Mathematical, Computational and Modeling Sciences Center, Arizona State University, Tempe, AZ, 85287, USA; 2 School of Life Sciences, Arizona State University, Tempe, AZ, 85287, USA; 3 School of Mathematical and Statistical Sciences, Arizona State University, Tempe, AZ, 85287, USA; 4 School of Human Evolution and Social Change, Arizona State University, Tempe, AZ, 85287, USA

## 

Neurons receive input from thousands of excitatory and inhibitory synapses, but they are not merely passive receivers and transmitters of information. The intrinsic properties of neurons, and more specifically the collection of ion channels expressed, influence a cell's excitability and integration processes. The role of different voltage-dependent transient (A-type) and persistent (delayed rectifier) potassium currents in shaping the activity of neurons in response to synaptic input is not well understood. We developed a biophysical model incorporating realistic synaptic input [1] to investigate the specific roles played by distinct A-type potassium channels encoded by different genes, namely *Shaker*/Kv1 and *Shal*/Kv4. We find that these two channel types, which are functionally distinguished by their inactivation properties, exert different control mechanisms on the transitions between rest and repetitive spiking in neurons. Bifurcation analysis is used to characterize the influence of each channel and the overall behavior of the membrane as a function of the relative presence of the two A-type channels. We found that the influence of each channel type depends on the resting potential of the neuron, suggesting that modulation and/or network states may shift their relative contribution. For example, at a particular membrane potential, Shal/Kv4 channels are more likely to mediate delays to first spike in response to excitatory input. Overall, this work increases our understanding of the interaction between intrinsic properties and synaptic input in creating neuronal response profiles. Further, we provide a link between relative gene expression and bifurcation structures in dynamical systems modeling neuronal membranes.
